# Effects of acute exposure to low-dose radiation on the characteristics of human bone marrow mesenchymal stromal/stem cells

**DOI:** 10.1186/s41232-017-0049-2

**Published:** 2017-09-01

**Authors:** Aya Fujishiro, Yasuo Miura, Masaki Iwasa, Sumie Fujii, Noriko Sugino, Akira Andoh, Hideyo Hirai, Taira Maekawa, Tatsuo Ichinohe

**Affiliations:** 1 Department of Transfusion Medicine and Cell Therapy, Kyoto University Hospital, 54 Kawaharacho, Shogoin, Sakyo-ku, Kyoto, 606-8507 Japan; 2 Division of Gastroenterology and Hematology, Department of Medicine, Shiga University of Medical Science, Setatsukinowacho, Otsu, Shiga 520-2192 Japan; 3 Department of Hematology/Oncology, Graduate School for Medicine, Kyoto University, 54 Kawaharacho, Shogoin, Sakyo-ku, Kyoto, 606-8507 Japan; 4 Department of Hematology and Oncology, Research Institute for Radiation Biology and Medicine, Hiroshima University, 1-2-3 Kasumi, Minamiku, Hiroshima, 734-8553 Japan

**Keywords:** Bone marrow mesenchymal stromal/stem cells, Low-dose irradiation, Human

## Abstract

**Background:**

In recent years, increasing attention has been paid to the effects of low-dose irradiation on human health. We examined whether low-dose irradiation affected the functions of mesenchymal stromal/stem cells (MSCs), which are tissue/organ-supportive stem cells, derived from bone marrow (BM).

**Methods:**

Normal human BM-MSCs from five healthy individuals were used in this study. Culture-expanded BM-MSCs were exposed to 0.1 gray (Gy) of γ-radiation (Cesium-137) at a rate of 0.8 Gy/min (Ir-MSCs), and their expansion, multi-differentiation, and hematopoiesis-supportive capabilities were investigated.

**Results:**

The expansion of BM-MSCs was transiently delayed after low-dose γ-irradiation compared with that of non-irradiated BM-MSCs (non-Ir-MSCs) in two out of five lots. Adipogenic and osteogenic differentiation capabilities were not significantly affected by low-dose irradiation, although one lot of BM-MSCs tended to have transiently reduced differentiation. When human BM hematopoietic stem/progenitor cells (HPCs) were co-cultured with Ir-MSCs, the generation of CD34^+^CD38^+^ cells from HPCs was enhanced compared with that in co-cultures with non-Ir-MSCs in two out of five lots. The mRNA expression level of interleukin (IL)-6 was increased and those of stem cell factor (SCF) and fms-related tyrosine kinase 3 ligand (Flt3L) were decreased in the affected lots of Ir-MSCs. In the other three lots of BM-MSCs, a cell growth delay, enhanced generation of CD34^+^CD38^+^ cells from HPCs in co-culture, and a combination of increased expression of IL-6 and decreased expression of SCF and Flt3L were not observed. Of note, the characteristics of these affected Ir-MSCs recovered to a similar level as those of non-Ir-MSCs following culture for 3 weeks.

**Conclusions:**

Our results suggest that acute exposure to low-dose (0.1 Gy) radiation can transiently affect the functional characteristics of human BM-MSCs.

## Background

Evaluation of the effects of low-dose irradiation is a medical and social issue for the health of patients who undergo computed tomography (CT) imaging for diagnosis, medical staff working in close proximity to radioisotopes, engineers working in nuclear power plants, and survivors of nuclear accidents such as the Fukushima Daiichi Nuclear Power Plant accident in 2011. The deterministic effects of radiation influence the risk of diseases apart from cancers [1], i.e., there is no influence on the risk of these diseases by exposure to low-dose radiation of equal to or less than the threshold dose of 0.1 gray (Gy). However, recent epidemiological studies suggest that such low-dose irradiation increases the relative risk of ischemic or non-ischemic heart diseases, cerebrovascular disease, and cataracts [2, 3]. Although not definitive, low-dose irradiation might affect the risk of a variety of non-malignant diseases as well as malignant diseases such as hematological malignancies [4].

In bone marrow (BM), there are two different types of stem/progenitor cells. With respect to hematopoiesis, hematopoietic stem/progenitor cells (HPCs) produce mature blood cells, and mesenchymal stromal/stem cells (MSCs) support this [5]. The influence of exposure to low-dose radiation on human HPCs has been investigated, and generation of both immature and mature hematopoietic cells from human HPCs is compromised [6, 7]. On the other hand, the influence of exposure to low-dose radiation on MSCs is not known, although MSCs help to maintain the balance of bone/hematopoietic tissues as tissue-specific stem/progenitor cells in BM.

In this study, we examined whether low-dose irradiation affected the expansion, differentiation, and hematopoiesis-supportive capability of human BM-MSCs.

## Methods

### Reagents and antibodies

Stem cell factor (SCF), interleukin (IL)-3, and fms-related tyrosine kinase 3 ligand (Flt3L) were purchased from Wako Pure Chemical Industries (Osaka, Japan). Thrombopoietin (TPO) was provided by Kyowa Hakko Kirin (Tokyo, Japan). A phycoerythrin (PE)-conjugated mouse anti-CD34 antibody (clone 563) and PE- or fluorescein isothiocyanate-conjugated mouse anti-CD45 antibodies (clone HI30) were purchased from BD Pharmingen (San Diego, CA). Allophycocyanin-conjugated mouse anti-CD38 (clone HIT2) and anti-CD45 (clone 2D1) antibodies and PE-conjugated mouse anti-CD14 (clone 61D3), anti-CD19 (clone SJ25C1), anti-CD73 (clone AD2), anti-CD90 (clone 5E10), and anti-CD105 (clone SN6) antibodies were from eBioscience (San Diego, CA).

### Isolation, expansion, and γ-irradiation of BM-MSCs

BM samples from healthy adult volunteers were purchased from AllCells (Emeryville, CA). BM-MSCs were isolated based on our previously published method [8, 9]. A single-cell suspension of 1 × 10^7^ BM mononuclear cells (MNCs) was seeded into T-75 culture flasks, and adherent cells were cultured in advanced-minimal essential medium (Invitrogen, Carlsbad, CA) supplemented with 5% fetal bovine serum (Invitrogen), 100 μM ascorbic acid (Wako Pure Chemical Industries), 2 mM L-glutamine, 100 U/mL penicillin, and 100 μg/mL streptomycin (all from Gibco, Carlsbad, CA). Primary cultures were passaged to disperse the colony-forming cells (passage 1). Cells at passage 3 were used as BM-MSCs in this study. Prior to experiments, the surface antigen profiles of CD14, CD19, CD34, CD45, CD73, CD90, and CD105 were examined by flow cytometric analysis to confirm that these cells expressed mesenchymal stem cell markers but did not express hematopoietic cell markers [10]. BM-MSCs were exposed to 0.1 Gy γ-radiation (Cesium-137) at a rate of 0.8 Gy/min using a Gammacell Irradiator (Best Theratronics, Ontario, Canada). Unless specified, γ-irradiated BM-MSCs (Ir-MSCs) were applied to the subsequent experiments at 24 h after irradiation (early phase). In some experiments, Ir-MSCs were cultured for a further 2–3 weeks after γ-irradiation and then applied to the subsequent experiments (late phase). To examine the effect of high-dose γ-radiation as a positive control, BM-MSCs were exposed to 4 Gy γ-radiation (H-MSCs) and were applied to the subsequent experiments. The study protocol was approved by the ethics committee of Kyoto University. For the cell expansion assay, BM-MSCs were seeded at a density of 5 × 10^4^ cells/10 cm dish and γ-irradiated, and then the number of cells was counted by 0.5% trypan blue staining weekly until they became confluent.

### Microarray analysis

Total RNA was isolated from Ir-MSCs and BM-MSCs that had not been exposed to 0.1 Gy γ-radiation (non-Ir-MSCs) using an RNeasy Mini Kit (Qiagen, Hilden, Germany). The RNA integrity of all samples was evaluated using an Agilent 2100 Bioanalyzer (Agilent Technologies, Santa Clara, CA) and an Agilent RNA 6000 Nano Kit (Agilent Technologies). A total of 150 ng RNA extracted from each sample was used to synthesize complementary DNA (cDNA) and complementary RNA (cRNA), and cRNA was labeled with Cyanine3. This was performed using a Low Input Quick Amp Labeling Kit, One-Color (Agilent Technologies). Hybridization was performed using a SurePrint G3 Human GE Microarray Kit (Agilent Technologies). Scanning and digitization of the microarray were performed using an Agilent Microarray Scanner G2565CA (Agilent Technologies) and Agilent Feature Extraction 11.0.1.1 (Agilent Technologies), respectively. Data normalization and expression analysis were performed using GeneSpring software version 13.1 (Agilent Technologies). Genes that were significantly up or downregulated in Ir-MSCs compared with non-Ir-MSCs were further analyzed by Gene Ontology (GO) classification, pathway analysis (Single Experiment Analysis [SEA] using Wikipathways; http://www.wikipathways.org), and Gene Set Enrichment Analysis ([GSEA]; GSEA v2.0.14 software, http://www.broadinstitute.org/gsea/index.jsp) [11–18]. Microarray data were submitted to the Gene Expression Omnibus of NCBI under the accession number GSE97368.

### In vitro differentiation assays of BM-MSCs

To induce osteogenic differentiation of BM-MSCs, osteogenesis-inducing cocktails of 100 μM ascorbic acid, 1.8 mM potassium dihydrogen phosphate (Sigma-Aldrich, St. Louis, MO), and 100 nM dexamethasone (Sigma-Aldrich) were added to the culture media. Mineralization was evaluated by 1% Alizarin Red S staining after 6 weeks of osteogenesis-inducing culture. To induce adipogenic differentiation of BM-MSCs, 0.5 mM isobutyl-methylxanthine, 60 μM indomethacin, 0.5 μM hydrocortisone, and 10 μg/mL insulin (all from Sigma-Aldrich) were added to the culture media. Oil Red O staining was used to assess lipid-laden fat cells after 3 weeks of adipogenesis-inducing culture. The Alizarin Red S-stained area and the number of Oil Red O^+^ cells were quantitated as previously described [9, 19]. Images were acquired using an Axiovert 40C microscope (Carl Zeiss, Oberkochen, Germany). In some experiments, expression of the adipogenic gene fatty acid-binding protein 4 (FABP4) and the osteogenic gene runt-related transcription factor 2 (Runx2) in H-MSCs was assessed by quantitative real-time PCR.

### Co-culture assays of CD34^+^ HPCs and BM-MSCs

Human CD34^+^ HPCs were isolated from BM-MNCs using anti-CD34 immunomagnetic microbeads (Miltenyi Biotec, Bergisch Gladbach, Germany). BM-MSCs (5 × 10^4^) were seeded into a 24-well culture plate and exposed to 0.1 Gy γ-radiation. Then, 0.6 × 10^3^ HPCs were applied to the BM-MSCs and co-cultured in StemSpan Serum-Free Expansion Medium (STEMCELL Technologies, Vancouver, Canada) supplemented with 100 ng/mL SCF, 100 ng/mL Flt3L, 50 ng/mL TPO, and 20 ng/mL IL-3. After 10 days of co-culture, the number and surface marker expression of hematopoietic cells were examined by flow cytometric analysis.

### Quantitative real-time PCR

Total RNA was extracted using the QIAamp RNA Blood Mini Kit (Qiagen) and subjected to reverse transcription. The 10 μL PCR mixture contained Taqman Fast Universal PCR master mix (Applied Biosystems, Carlsbad, CA), cDNA, primer pairs, and the Taqman probe (Universal Probe Library). cDNA was amplified with the Step One Plus Real-Time PCR System (Applied Biosystems) using the following parameters: 95 °C for 20 s, followed by 40 cycles of 95 °C for 1 s and 60 °C for 20 s. Glyceraldehyde-3-phosphate dehydrogenase was used as an internal control to normalize any loading differences. The primer sets and universal probes used are listed in Table 1.

**Table 1:** List of primer sets and universal probes for quantitative real-time PCR.

Gene	Forward primer (5′–3′)	Reverse primer (5′–3′)	Universal probe (#)
Ang-1	gacagatgttgagacccaggta	tgcttctctagcttgtaggtgga	67
Flt3L	ggccgaaatgacagtgct	agcagcaggaggagataggtt	1
GAPDH	agccacatcgctcagacac	gcccaatacgaccaaatcc	60
IL-6	gatgagtacaaaagtcctgatcca	ctgcagccactggttctgt	40
Jag-1	ggcaacaccttcaacctca	gcctccacaagcaacgtatag	28
LIF	tgccaatgccctctttattc	gtccaggttgttggggaac	26
SCF	gcgctgcctttccttatg	ccttcagttttgacgagagga	68
FABP4	ccaccataaagagaaaacgagag	gtggaagtgacgcctttcat	77
Runx2	ctaccaccccgctgtcttc	aaaaagggcccagttctga	4

*Ang-1* angiopoietin-1, *Flt3L* fms-related tyrosine kinase 3 ligand, *GAPDH* glyceraldehyde-3-phosphate dehydrogenase, *IL-6* interleukin-6, *Jag-1* jagged-1, *LIF* leukemia inhibitory factor, *SCF* stem cell factor, *FABP4* fatty acid-binding protein 4, *Runx2* runt-related transcription factor 2

### Statistical analysis

The unpaired Student’s *t* test was used for analysis, unless otherwise indicated. Data in bar graphs indicate the mean ± standard deviation (SD). Statistical significance is expressed as follows: *, *p* < 0.05; **, *p* < 0.01; n.s., not significant.

## Results

### Expansion of Ir-MSCs

**Fig. 1:**
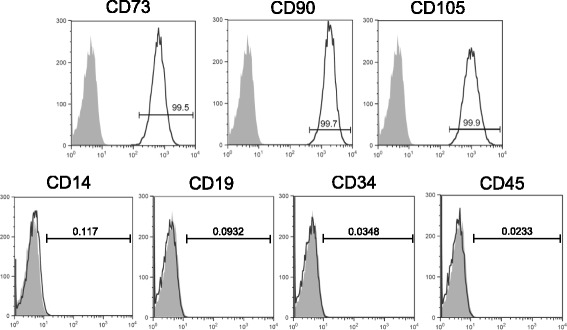
Surface marker expression of human BM-MSCs. Flow cytometric analysis shows that lot A of BM-MSCs is positive for mesenchymal stem cell-associated surface markers (CD73, CD90, and CD105) and is negative for hematopoietic cell-associated surface markers (CD14, CD19, CD34, and CD45). *Filled histograms* indicate control staining. *Numbers* in each histogram indicate the percentage of cells. The same surface marker expression profiles were confirmed in lots B–E of BM-MSCs

**Fig. 2:**
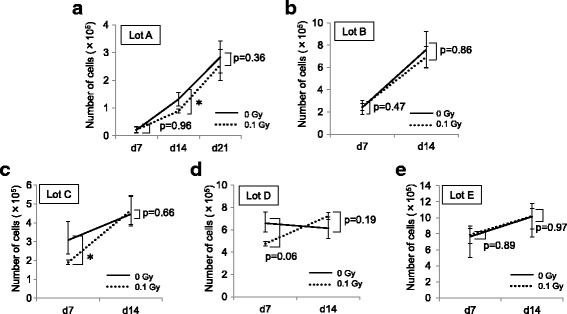
Expansion of Ir-MSCs. (**a**–**e**) BM-MSCs (lots A–E) were exposed to (Ir-MSCs, *dotted lines*) or not exposed to (non-Ir-MSCs, *solid lines*) 0.1 Gy γ-radiation on day 0. They were then cultured until confluent. The number of viable cells was counted weekly after γ-irradiation using 0.5% trypan blue. Data are mean values ± SD. *n* = 5 per group (**a**–﻿**c**) or *n* = 3 per group (**d**, **e**). *, *p* < 0.05

**Fig. 3:**
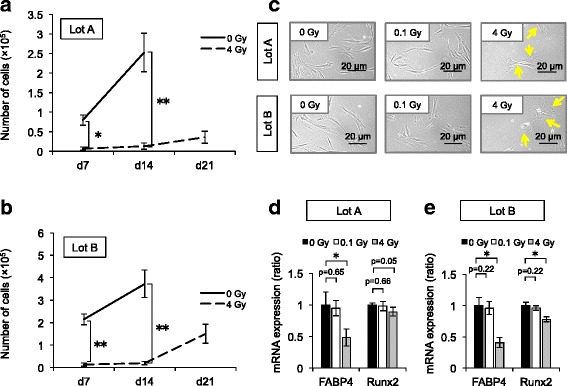
Characteristics of H-MSCs. BM-MSCs (lots A and B) were exposed to 4 Gy (H-MSCs) or 0.1 Gy (Ir-MSCs) γ-radiation, or were not exposed to γ-radiation (non-Ir-MSCs). (**a**, **b**) Expansion of lots A and B of H-MSCs (*dashed lines*) and non-Ir-MSCs (*solid lines*). The number of viable cells was counted weekly after γ-irradiation using 0.5% trypan blue. (**c**) Morphology of lots A and B of H-MSCs, Ir-MSCs, and non-Ir-MSCs. Representative phase contrast images are shown. *Yellow arrows* indicate H-MSCs. Bars, 20 μm. (**d**, **e**) Expression of adipogenic and osteogenic markers in lots A and B of H-MSCs (*gray bars*), Ir-MSCs (*open bars*), and non-Ir-MSCs (*solid bars*), as assessed by quantitative real-time PCR. Expression in H-MSCs and Ir-MSCs is shown relative to that in non-Ir-MSCs. Data are mean values ± SD (**a**, **b**, **d**, **e**). *n* = 4 per group (**a**, **d**) or *n* = 5 per group (**b**, **e**). *, *p* < 0.05; **, *p* < 0.01

Five lots of human BM-MSCs (lots A–E) were tested in this study. These cells were positive for mesenchymal stem cell-associated markers including CD73, CD90, and CD105 and were negative for hematopoietic cell-associated markers including CD14, CD19, CD34, and CD45 (Fig. 1). Each lot of BM-MSCs was exposed to 0.1 Gy γ-radiation, and then their expansion was evaluated. In lot A, the number of Ir-MSCs on day 14 after γ-irradiation was lower than the number of non-Ir-MSCs (Fig. 2a). On the other hand, the numbers of Ir-MSCs and non-Ir-MSCs did not differ in lot B (Fig. 2b). With regard to the other three lots of BM-MSCs, the number of Ir-MSCs on day 7 after γ-irradiation was lower than the number of non-Ir-MSCs in one lot (lot C, Fig. 2c), and there was no difference between the numbers of Ir-MSCs and non-Ir-MSCs in the other two lots (lots D and E, Fig. 2d, e). Of note, in lots A and C, the number of Ir-MSCs recovered to a level similar to the number of non-Ir-MSCs by culture for 3 weeks after γ-irradiation (Fig. 2a, c). Therefore, BM-MSCs were divided into two groups based on their expansion response to acute exposure to low-dose γ-radiation. One group included BM-MSCs whose expansion was not affected by exposure to low-dose γ-radiation. The other group included BM-MSCs that showed a transient expansion delay upon exposure to low-dose γ-radiation. In lots A and B, the number of H-MSCs was consistently lower than the number of non-Ir-MSCs (Fig. 3a, b). H-MSCs changed their morphology from a spindle shape to an enlarged and irregular shape, whereas the morphology of Ir-MSCs was comparable to that of non-Ir-MSCs (Fig. 3c).

**Fig. 4:**
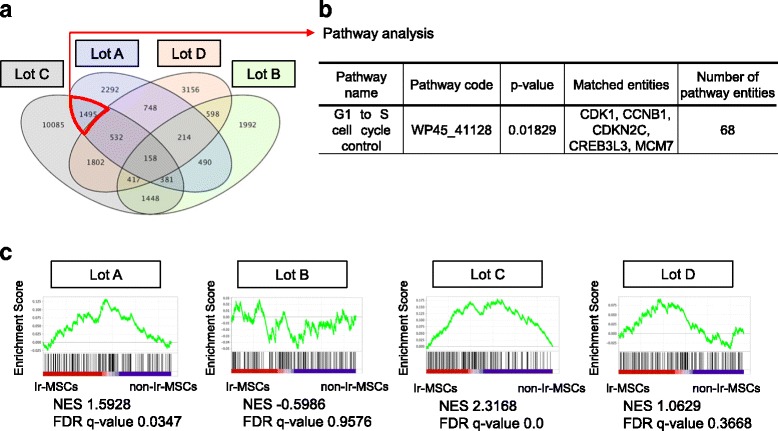
Microarray analysis of Ir-MSCs. (**a**) Venn diagram of the number of genes whose expression levels were downregulated in lots A, B, C, and D of Ir-MSCs compared with non-Ir-MSCs. The *purple*, *green*, *gray*, and *orange ellipses* represent genes in lots A, B, C, and D, respectively. (**b**) Pathway analysis was performed of 1495 genes that were downregulated in both lots A and C of Ir-MSCs, but not in lot B or D of Ir-MSCs. The pathway “G1 to S cell cycle control” was significantly enriched. (**c**) GSEA of genes from lots A, B, C, and D of Ir-MSCs and non-Ir-MSCs using the gene set AMUNDSON_POOR_SURVIVAL_AFTER_GAMMA_RADIATION_2G. *NES* normalized enrichment score, *FDR* false discovery rate

We performed microarray analysis using RNA extracted from Ir-MSCs and non-Ir-MSCs. The number of genes that were upregulated in Ir-MSCs relative to non-Ir-MSCs (fold change >1.2) was 8290, 5510, 6252, and 8383 in lots A, B, C, and D, respectively. Among them, 783 genes were commonly upregulated only in lots A and C of Ir-MSCs, which showed an expansion delay after γ-irradiation. SEA of these 783 genes identified three pathways with significant changes; however, they were not associated with well-known functions of MSCs. The number of genes that were downregulated in Ir-MSCs relative to non-Ir-MSCs (fold change >1.2) was 6310, 5698, 16,318, and 7625 in lots A, B, C, and D, respectively (Fig. 4a). Among them, 1495 genes were commonly downregulated only in lots A and C. SEA of these 1495 genes identified seven pathways with significant changes, including a cell cycle-associated pathway (Fig. 4b). We also performed GSEA of genes from both Ir-MSCs and non-Ir-MSCs, and found that the gene set “AMUNDSON_POOR_SURVIVAL_AFTER_GAMMA_RADIATION_2G” was highly enriched in lots A and C of Ir-MSCs, but not in lot B or D of Ir-MSCs (Fig. 4c). This gene set consists of genes that are upregulated in the 60 damaged cell lines of the National Cancer Institute Anticancer Drug Screen (NCI-60) after genotoxic stress induced by 2 Gy γ-irradiation [20]. This result suggests that stress in Ir-MSCs was comparable to that induced by 2 Gy γ-irradiation.

### Adipogenic and osteogenic differentiation capabilities of Ir-MSCs

**Fig. 5:**
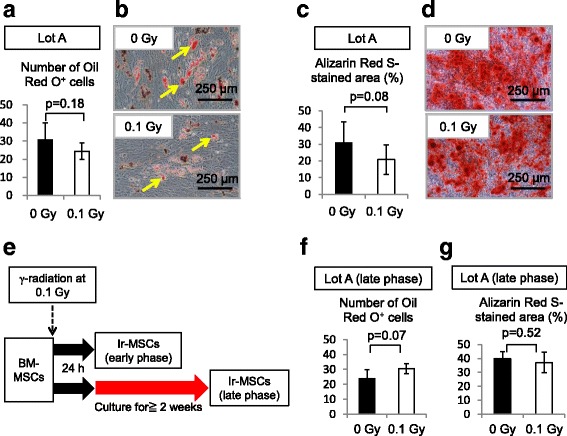
Adipogenic and osteogenic differentiation of Ir-MSCs. (**a**) Quantitative measurement of the adipogenic differentiation of lot A of BM-MSCs that were exposed to (*white bar*) or not exposed to (*black bar*) 0.1 Gy γ-radiation, as assessed by Oil Red O staining. (**b**) Representative images are shown. *Yellow arrows* indicate lipid-laden fat cells. *Bars* 250 μm. (**c**) Quantitative measurement of the osteogenic differentiation of lot A of BM-MSCs that were exposed to (*white bar*) or not exposed to (*black bar*) 0.1 Gy γ-radiation, as assessed by Alizarin Red S staining. (**d**) Representative images are shown. *Bars* 250 μm. (**e**–**g**) Adipogenic and osteogenic differentiation of Ir-MSCs at the late phase. (**e**) Schema of culture of BM-MSCs after γ-irradiation. (**f**, **g**) Quantitative measurement of the adipogenic (**f**) and osteogenic (**g**) differentiation of lot A of BM-MSCs that were exposed to (*white bars*) or not exposed to (*black bars*) 0.1 Gy γ-radiation and then cultured for a further 3 weeks (Ir-MSCs at late phase), as assessed by Oil Red O staining and Alizarin Red S staining, respectively. (**a**, **c**, **f**, **g**) Data are mean values ± SD. *n* = 5 per group

**Fig. 6:**
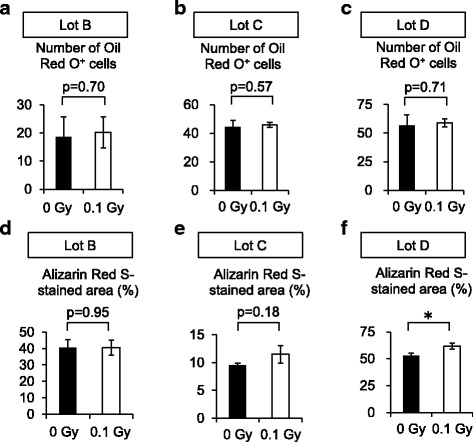
Adipogenic and osteogenic differentiation of three different lots of Ir-MSCs. (**a**–**c**) Quantitative measurement of the adipogenic differentiation of three different lots of BM-MSCs (lots B, C, and D) that were exposed to (*white bars*) or not exposed to (*black bars*) 0.1 Gy γ-radiation, as assessed by Oil Red O staining. (**d**–**f**) Quantitative measurement of the osteogenic differentiation of three different lots of BM-MSCs (lots B, C, and D) that were exposed to (*white bars*) or not exposed to (*black bars*) 0.1 Gy γ-radiation, as assessed by Alizarin Red S staining. Lot E of BM-MSCs was unavailable for this analysis due to an inadequate number of cells. Data are mean values ± SD. *n* = 4 per group (**a**, **d**) or *n* = 3 per group (**b**, **c**, **e**, **f**). *, *p* < 0.05

A multi-differentiation capability is one of the fundamental characteristics of human BM-MSCs. We examined the influence of acute exposure to 0.1 Gy γ-radiation on the adipogenic and osteogenic differentiation of BM-MSCs. Although not statistically significant, Ir-MSCs tended to show reduced adipogenic and osteogenic differentiation capabilities in lot A, as assessed by Oil Red O staining (Fig. 5a, b) and Alizarin Red S staining (Fig. 5c, d), respectively. In the other lots of BM-MSCs, there was no difference in the level of fat deposition between adipogenically differentiated Ir-MSCs and non-Ir-MSCs (Fig. 6a–c). With regard to the level of mineralization, there was no difference between osteogenically differentiated Ir-MSCs and non-Ir-MSCs in all lots except for lot D (Fig. 6d–f). We performed the same experiments using lot A of BM-MSCs that had been cultured for a further 2 weeks after γ-irradiation (Fig. 5e, late phase). Their differentiation capabilities were similar to those of non-Ir-MSCs (Fig. 5f, g). We examined expression of the adipogenic gene FABP4 and the osteogenic master gene Runx2 in lots A and B of H-MSCs. Their expression was low in both lots of H-MSCs compared with that in non-Ir-MSCs, whereas it was comparable in Ir-MSCs and non-Ir-MSCs (Fig. 3d, e).

### Hematopoiesis-supportive capability of Ir-MSCs

**Fig. 7:**
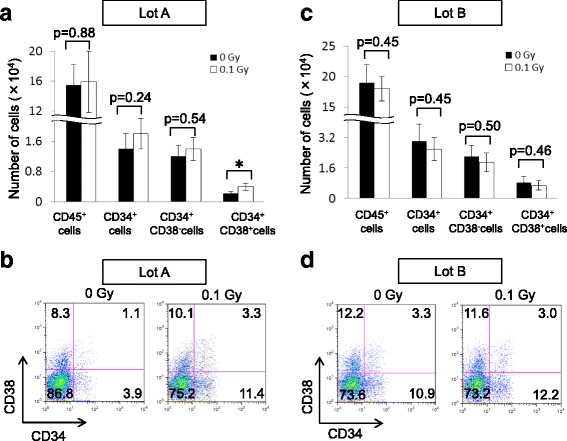
Co-culture of HPCs with Ir-MSCs. Flow cytometric analysis showing the generation of hematopoietic cells from HPCs after 10 days of co-culture with lot ﻿A (**a**, **b**) and lot B (**c**, **d**) of BM-MSCs that were exposed to (*white bars*) or not exposed to (*black bars*) 0.1 Gy γ-radiation. The numbers of CD45^+^ cells, CD34^+^ cells, CD34^+^CD38^−^ cells, and CD34^+^CD38^+^ cells are shown in (**a**, **c**). Data are mean values ± SD. *n* = 5 per group. *, *p* < 0.05. Representative dot plots of CD34 versus CD38 expression are shown in (**b**, **d**). *Numbers* in each box indicate the percentage of cells

**Fig. 8:**
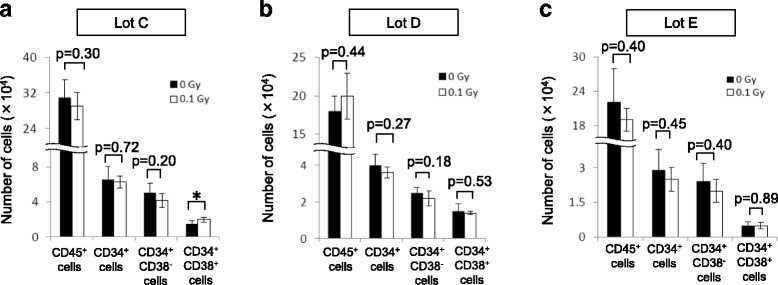
Generation of hematopoietic cells from HPCs in co-culture with Ir-MSCs. (**a**–**c**) The numbers of CD45^+^ cells, CD34^+^ cells, CD34^+^CD38^−^ cells, and CD34^+^CD38^+^ cells in co-culture with three different lots of BM-MSCs (lots C, D, and E) that were exposed to (*white bars*) or not exposed to (*black bars*) 0.1 Gy γ-radiation are shown. Data are mean values ± SD. *n* = 5 per group. *, *p* < 0.05

We examined whether the capability of BM-MSCs to support hematopoiesis was affected by low-dose γ-irradiation. Twenty-four hours after γ-irradiation, Ir-MSCs were co-cultured with CD34^+^ HPCs in vitro. In lot A, the generation of CD45^+^ cells, total CD34^+^ cells, and CD34^+^CD38^−^ cells from HPCs did not significantly differ between co-cultures with Ir-MSCs and co-cultures with non-Ir-MSCs. However, the generation of CD34^+^CD38^+^ cells was enhanced in co-cultures with Ir-MSCs compared with that in co-cultures with non-Ir-MSCs (Fig. 7a, b). In lot B, the generation of CD34^+^CD38^+^ cells as well as that of CD45^+^ cells, total CD34^+^ cells, and CD34^+^CD38^−^ cells in co-cultures with Ir-MSCs was similar to that in co-cultures with non-Ir-MSCs (Fig. 7c, d). When the same experiments were performed using the other three lots of BM-MSCs (lots C, D, and E), the enhanced generation of CD34^+^CD38^+^ cells was observed in lot C, but not in lots D and E (Fig. 8a–c). Thus, BM-MSCs were divided into two groups based on their response to acute exposure to low-dose γ-radiation. One group included BM-MSCs whose capability to support hematopoietic cell generation from HPCs in co-cultures was not affected by exposure to 0.1 Gy γ-radiation. The other group included BM-MSCs whose hematopoiesis-supportive capability was affected by such γ-irradiation.

### Increased IL-6 expression and decreased SCF and Flt3L expression in Ir-MSCs that show an altered hematopoiesis-supportive capability

**Fig. 9:**
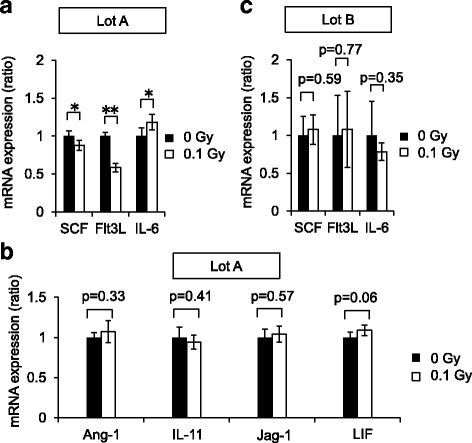
Expression of hematopoiesis-associated molecules in Ir-MSCs. (**a**, **b**) mRNA expression of various hematopoiesis-associated molecules in lot A of BM-MSCs that were exposed to (Ir-MSCs, *white bars*) or not exposed to (non-Ir-MSCs, *black bars*) 0.1 Gy γ-radiation, as assessed by quantitative real-time PCR analysis. Molecules whose expression levels differed between Ir-MSCs and non-Ir-MSCs are shown in (**a**), whereas those whose expression levels did not differ between Ir-MSCs and non-Ir-MSCs are shown in (**b**). Expression in Ir-MSCs is shown relative to that in non-Ir-MSCs. Data are mean values ± SD. *n* = 5 per group. *, *p* < 0.05; **, *p* < 0.01. (**c**) mRNA expression of molecules affected in lot A of BM-MSCs was examined in lot B of BM-MSCs that were exposed to (Ir-MSCs, *white bars*) or not exposed to (non-Ir-MSCs, *black bars*) 0.1 Gy γ-radiation, as assessed by quantitative real-time PCR analysis. Data are mean values ± SD. *n* = 5 per group

We next explored the expression levels of hematopoiesis-associated molecules in Ir-MSCs. In lot A, the mRNA expression level of IL-6 was higher and those of SCF and Flt3L were lower in Ir-MSCs than in non-Ir-MSCs (Fig. 9a). With regard to other hematopoiesis-associated molecules, the mRNA expression levels of angiopoietin-1 (Ang-1), jagged-1 (Jag-1), leukemia inhibitory factor (LIF), and IL-11 did not differ between Ir-MSCs and non-Ir-MSCs (Fig. 9b). In lot B, the mRNA expression levels of IL-6, SCF, and Flt3L in Ir-MSCs were similar to those in non-Ir-MSCs (Fig. 9c). When the same experiments were performed using the other three lots of BM-MSCs, there was an increase in mRNA expression of IL-6 in combination with a decrease in mRNA expression of SCF and Flt3L in lot C (Table 2). On the other hand, this combined alteration in mRNA expression of IL-6, SCF, and Flt3L was not observed in lots D and E (Table 2).

**Table 2:** Hematopoiesis-associated characteristics of five lots of BM-MSCs that were exposed to 0.1 Gy γ-radiation.

		Lot of BM-MSCs				
Phase of BM-MSCs after low-dose γ-irradiation	Characteristics of low-dose γ-irradiated BM-MSCs	A	C	B	D	E
Early phase (24 hours)	Enhanced generation of CD34^+^CD38^+^ cells from HPCs	+	+	−	−	−
	Increased expression of IL-6/Decreased expression of SCF and Flt3L	+	+	−	−	−
Late phase (3 weeks)	Enhanced generation of CD34^+^CD38^+^ cells from HPCs	−	−	n.t.	n.t.	n.t.
	Increased expression of IL-6/Decreased expression of SCF and Flt3L	−	−	n.t.	n.t.	n.t.

*BM-MSCs* bone marrow mesenchymal stromal/stem cells, *HPCs* hematopoietic stem/progenitor cells, *IL-6* interleukin-6, *SCF* stem cell factor, *Flt3L* fms-related tyrosine kinase 3 ligand, *n.t.* not tested

### Recovery of the altered hematopoiesis-associated characteristics of Ir-MSCs

Finally, we investigated whether the altered hematopoiesis-associated characteristics of lots A and C of Ir-MSCs recovered. BM-MSCs were cultured for a further 3 weeks after γ-irradiation (Fig. 5e, late phase). In both lots A and C, the mRNA expression levels of IL-6, SCF, and Flt3L in Ir-MSCs at the late phase were similar to those in non-Ir-MSCs. In addition, when Ir-MSCs at the late phase were co-cultured with CD34^+^ HPCs, the generation of CD34^+^CD38^+^ cells was similar to that in co-cultures with non-Ir-MSCs (Table 2).

## Discussion

The stochastic effects of irradiation on the risk of cancers including hematological malignancies are well established [4]. Diagnostic X-rays can increase the risk of cancer [21], and chromosome translocation induced by a single CT scan has been demonstrated at the cellular level [22]. On the other hand, 0.1 Gy is the threshold of the deterministic effects of irradiation, and low-dose irradiation of equal to or less than 0.1 Gy is considered to have no influence on the risk of diseases apart from malignancies. However, this dose of irradiation can induce somatic mutations or genomic mutations involved in cancer development [23, 24]. Thus, studies to evaluate the effects of low-dose irradiation on tissue or organ functions are required [25, 26].

In the microarray analysis presented in this study, cell cycle-associated genes were downregulated in the group of BM-MSCs that showed an expansion delay upon exposure to 0.1 Gy γ-radiation. Previous studies indicate that low-dose irradiation induces apoptosis and cell cycle arrest in BM-MSCs [27, 28]. γ-radiation at a dose of 0.1 Gy might delay the cell cycle in Ir-MSCs, resulting in the delayed expansion of MSCs. In addition, a gene set consisting of genes that are upregulated in the damaged NCI-60 cell lines after 2 Gy irradiation [20] was highly enriched in the lots of BM-MSCs that showed an expansion delay upon exposure to 0.1 Gy γ-radiation. BM-MSCs could be affected by low-dose γ-irradiation, even 0.1 Gy, as well as by high-dose γ-irradiation.

DNA damage occurs upon a single irradiation of 0.1 Gy [27–29]. DNA damage following low-dose X-ray exposure was demonstrated in human gingival MSCs by the formation of phosphorylated histone H2AX and phosphor-S1981 ATM foci [28]. The altered expression of hematopoiesis-associated molecules in Ir-MSCs that was observed in the present study might be affected by DNA damage. However, microarray analysis did not identify enrichment of a gene set related to DNA damage after 0.1 Gy γ-irradiation. The DNA repair process starts within several minutes after DNA damage and ends after several hours [30]. We speculate that DNA repair was almost complete when total RNA was extracted from Ir-MSCs and non-Ir-MSCs in the present study.

In this study, there were two patterns of the early response to acute exposure to low-dose γ-irradiation (0.1 Gy) in terms of the hematopoiesis-supportive characteristics of BM-MSCs, namely, BM-MSCs whose supportive capability to generate CD34^+^CD38^+^ cells from HPCs in co-culture was increased and BM-MSCs whose such characteristics were not affected by low-dose γ-irradiation. Upregulated IL-6 expression and downregulated SCF and Flt3L expression were observed only in Ir-MSCs whose supportive capability to generate CD34^+^CD38^+^ cells from HPCs in co-culture was increased. These changes were not observed in Ir-MSCs whose characteristics were not affected by γ-irradiation. The difference in the response to γ-irradiation among multiple lots of BM-MSCs might be related to a difference in sensitivity to γ-irradiation. This is supported by our findings that multiple differentiation capabilities of Ir-MSCs were not substantially perturbed, regardless of whether their hematopoiesis-associated characteristics were affected. Alternatively, increased expression of IL-6 might reflect cellular senescence due to genotoxic stress in Ir-MSCs [31, 32]. IL-6 contributes to commitment of HPCs toward the myeloid lineage [33]. Therefore, alterations in the expression pattern of these hematopoiesis-associated molecules in Ir-MSCs contributed, at least partially, to the response to γ-irradiation.

In the present study, the increased supportive capability to generate CD34^+^CD38^+^ cells from HPCs in co-culture, along with upregulated IL-6 expression and downregulated SCF and Flt3L expression, and the delay in cell expansion were transient in the affected lots of human Ir-MSCs. Transient damage caused by low-dose irradiation has been reported in mouse BM-MSCs, in which the expansion speed of mouse BM-MSCs was reduced by irradiation at a dose of 0.5 J/cm^2^ for 16 and 33 s and was recovered by 72 h after irradiation, and nuclear alterations were not detected by 4,6-diamidino-2-phenylindole staining in irradiated mouse BM-MSCs [34]. Further studies are needed to elucidate the exact mechanism that underlies low-dose irradiation-induced functional changes in the characteristics of BM-MSCs.

## Conclusions

**Fig. 10:**
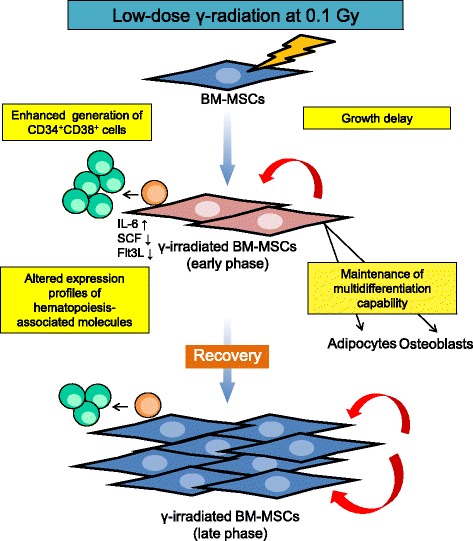
Graphical summary. Acute exposure to low-dose γ-radiation (0.1 Gy) could affect the functional characteristics of human BM-MSCs at the early phase. However, these changes were transient and recovered at the late phase

We demonstrated that acute exposure to low-dose (0.1 Gy) γ-radiation could affect the functional characteristics of human BM-MSCs including their hematopoiesis-supportive capability and expansion. These changes were transient, and the characteristics of Ir-MSCs recovered such that they were similar to those of non-Ir-MSCs (Fig. 10). Further research incorporating larger numbers of samples is warranted.

## Abbreviations

Ang-1: Angiopoietin-1
BM: Bone marrow
cDNA: Complementary DNA
cRNA: Complementary RNA
CT: Computed tomography
FABP4: Fatty acid-binding protein 4
Flt3L: Fms-related tyrosine kinase 3 ligand
GAPDH: Glyceraldehyde-3-phosphate dehydrogenase
GO: Gene Ontology
GSEA: Gene Set Enrichment Analysis
Gy: Gray
H-MSCs: BM-MSCs exposed to 4 Gy γ-radiation
HPCs: Hematopoietic stem/progenitor cells
IL: Interleukin
Ir-MSCs: BM-MSCs exposed to 0.1 Gy γ-radiation
Jag-1: Jagged-1
LIF: Leukemia inhibitory factor
MNCs: Mononuclear cells
MSCs: Mesenchymal stromal/stem cells
n.s.: Not significant
NCI-60: The 60 cell lines of the National Cancer Institute Anticancer Drug Screen
non-Ir-MSCs: BM-MSCs not exposed to 0.1 Gy γ-radiation
PE: Phycoerythrin
Runx2: Runt-related transcription factor 2
SCF: Stem cell factor
SD: Standard deviation
SEA: Single Experiment Analysis
TPO: Thrombopoietin
